# Peroral pancreatoscopy-guided electrohydraulic lithotripsy for impacted stones complicated by pancreatic pleural effusion

**DOI:** 10.1055/a-2875-0155

**Published:** 2026-06-03

**Authors:** Tatsuya Kageyama, Arata Sakai, Yoshiyuki Harada, Takashi Kobayashi, Atsuhiro Masuda, Norimitsu Uza, Yuzo Kodama

**Affiliations:** 1 Division of Gastroenterology, Department of Internal Medicine 12885 Kobe UniversityKobeHyogo PrefectureJapan

Extracorporeal shock wave lithotripsy (ESWL) has been established as the first-line treatment for pancreatic stones in non-surgical therapy. 1​ More recently, peroral pancreatoscopy (POPS)-guided electrohydraulic lithotripsy (EHL) has been reported as effective for difficult pancreatic stones and may shorten the treatment course. 2​ ​3​ We present a case of impacted pancreatic stones complicated by pancreatic duct leakage and pleural effusion, successfully treated with POPS-guided EHL in a single session.

**Fig. 1:**
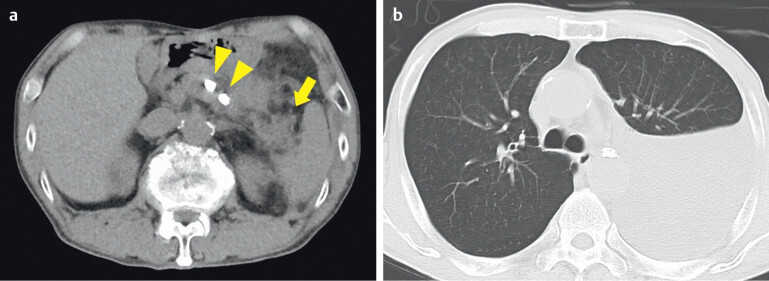
( **a** ) A computed tomography image showing two pancreatic stones in the pancreatic body and a peripancreatic fluid collection around the pancreatic tail. ( **b** ) A computed tomography image showing left-sided pleural effusion.

**Fig. 2:**
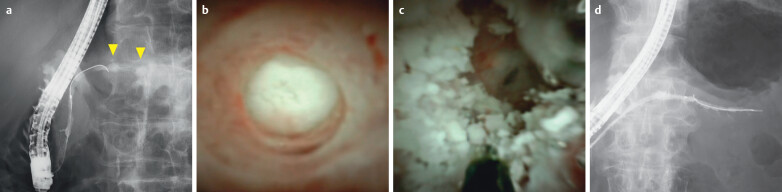
( **a** ) A fluoroscopic image showing two impacted stones in the pancreatic duct, resulting in failure of guidewire advancement into the distal duct. ( **b** ) A pancreatoscopic image showing an impacted pancreatic stone in the main pancreatic duct. ( **c** ) A pancreatoscopic image showing the successful fragmentation of a pancreatic stone after electrohydraulic lithotripsy (EHL). ( **d** ) A fluoroscopic image demonstrating opacification of the distal pancreatic duct after fragmentation of the impacted stones by pancreatoscopy-guided electrohydraulic lithotripsy (EHL).

A 68-year-old man with chronic pancreatitis presented with dyspnea and elevated pancreatic enzymes, with a serum amylase level of 1,480 U/L and a serum lipase level of 1,909 U/L. Computed tomography (CT) revealed two stones in the pancreatic body, accompanied by a peripancreatic fluid collection and left pleural effusion ( Fig. 1a and b ). Thoracentesis demonstrated an elevated amylase level in the pleural fluid of 52,060 U/L, consistent with pleural effusion secondary to pancreatic duct leakage. A chest tube was placed, and ERCP was performed for pancreatic duct drainage. Pancreatography revealed two impacted stones in the pancreatic body preventing guidewire advancement into the distal duct ( Fig. 2a ). A POPS (SpyGlass DS; Boston Scientific, Marlborough, MA, USA) was introduced into the main pancreatic duct (MPD), allowing the visualization of the pancreatic stone ( Fig. 2b ). Repeated EHL (AUTOLITH TOUCH; Northgate Technologies Inc., Elgin, IL, USA) achieved fragmentation of both the proximal and distal stones ( Fig. 2c ). This enabled distal MPD opacification and placement of a 5-Fr endoscopic nasopancreatic drainage tube ( Fig. 2d ). Repeat ERCP confirmed the resolution of ductal leakage, and residual stone fragments were extracted with a basket catheter. Follow-up CT demonstrated a marked reduction of the pancreatic stones and resolution of the pleural effusion, allowing chest tube removal and discharge ( Video 1 ).

**Video 1** Pancreatoscopy-guided electrohydraulic lithotripsy (EHL) for impacted pancreatic stones.


In this case, POPS-guided EHL was preferred over ESWL because immediate stone fragmentation was required to achieve prompt pancreatic duct drainage and control pancreatic duct leakage–associated pleural effusion.

Endoscopy_UCTN_Code_TTT_1AR_2AL
